# The association between Weight-adjusted-Waist Index (WWI) and cognitive function in older adults: a cross-sectional NHANES 2011–2014 study

**DOI:** 10.1186/s12889-024-19332-w

**Published:** 2024-08-08

**Authors:** Xichenhui Qiu, Jiahao Kuang, Yiqing Huang, Changning Wei, Xujuan Zheng

**Affiliations:** 1 Health Science Center, Shenzhen University, No. 1066, Xueyuan Avenue, Nanshan District, Shenzhen, Guangdong Province 518060 People’s Republic of China; 2 School of Tech X Academy, Shenzhen Polytechnic University, No. 7098, Liuxian Avenue Nanshan District, Shenzhen, Guangdong Province 518118 People’s Republic of China

**Keywords:** WWI, Weight-adjusted-Waist Index, Obesity, Cognitive function

## Abstract

**Background:**

The impact of obesity on cognitive function has engendered considerable interest. Weight-adjusted waist index (WWI) has emerged as a novel and innovative marker of obesity that reflects weight-independent abdominal obesity. However, the association between WWI and cognitive function remains unclear. To address this gap, the present study aims to explore the relationship between weight-adjusted waist index (WWI) and cognitive performance in older adults.

**Methods:**

We conducted a cross-sectional investigation using datasets from the National Health and Nutrition Examination Survey (NHANES) 2011–2014. The study included 3,472 participants (48.59% male, 51.41% female) of various races (Mexican American, Other Hispanic, Non-Hispanic White, Non-Hispanic Black, and Other), with a mean age of 69.95 years (SD = 6.94). Multivariate regression and smoothing curve fitting were used to investigate the linear and nonlinear relationship between WWI and cognitive performance in the following domains: learning and memory, verbal fluency, and processing speed, as measured by Consortium to Establish a Registry for Alzheimer’s Disease Word Learning subtest (CERAD-WL), Animal Fluency Test (AFT), and Digit Symbol Substitution Test (DSST), respectively. Subgroup analysis and interaction tests were conducted to examine the stability of this relationship across groups. Machine learning models based on random forests were used to analyze the predictive performance of WWI for cognitive function.

**Results:**

A total of 3,472 participants were included in the analysis. The results revealed significant negative associations between WWI and low scores on the CERAD-WL [-0.96 (-1.30, -0.62)], AFT [-0.77 (-1.05, -0.49)], and DSST [-3.67 (-4.55, -2.79)]. This relationship remained stable after converting WWI to a categorical variable. In addition, this significant negative association was more pronounced in men than women and diminished with advancing age. Non-linear threshold effects were observed, with correlations intensifying between WWI and CERAD-WL when WWI surpassed 12.25, AFT when WWI surpassed 11.54, and DSST when WWI surpassed 11.66.

**Conclusions:**

A higher WWI, indicating increased abdominal obesity, was associated with deficits in learning, memory, verbal fluency, and processing speed among older adults. These findings suggest that abdominal obesity may play a crucial role in cognitive decline in this population. The stronger relationship observed between WWI and cognition in men highlights the need for gender-specific considerations in interventions targeting abdominal obesity. The results demonstrate the importance of interventions targeting abdominal obesity to preserve cognitive performance in older adults.

**Supplementary Information:**

The online version contains supplementary material available at 10.1186/s12889-024-19332-w.

## Background

Obesity has long been associated with numerous health conditions, spanning cardiovascular diseases, chronic heart disease, hypertension, diabetes, stroke, and cancer [1–4]. The rising aging population gives rise to pressing concerns regarding cognitive health. The relationship between obesity and cognitive health is increasingly recognized. It has been reported that there is a negative correlation between obesity-related body measurements, such as body mass index (BMI) and waist circumference, and certain cognitive domains. For example, obesity is associated with impaired performance on explicit memory tasks. Language learning, measured by delayed recall and recognition of words, is affected in both higher and lower BMI populations [5, 6]. Similar impairments are also evident in visual pattern memory tasks [7]. Working memory abilities are also found to be impaired in overweight and obese young adults compared to healthy weight controls [8]. However, some studies have reported no differences in memory performance between obese and non-obese individuals [9]. Furthermore, there are notable impairments in cognitive domains unrelated to memory. For example, it has been reported that obese individuals exhibit impaired psychomotor abilities [5] and selective attention [10], although these findings are inconsistent [11, 12]. Performance on the Wisconsin Card Sorting Test, measuring executive functions related to concept formation and set-shifting, is also found to be diminished in the obese population compared to normal-weight comparison groups [13, 14]. Impaired performance in language fluency, memory, and comprehensive screening measures is also associated with obesity. Higher body mass index (BMI), waist circumference, and waist-to-hip ratio (WHR) are associated with poorer performance when measuring overall cognitive function [15].

While numerous studies have examined the relationship between obesity and cognitive function, their findings have frequently been inconsistent and ambiguous. Several studies have linked obesity with cognitive decline or even dysfunction in older adults [16–18]. However, newer research suggests there might be no increased risk of dementia due to obesity [19], and some even found a reduced risk among obese individuals [20, 21]. These discrepancies may arise from an undue reliance on BMI as the predominant measure of obesity [22]. Such ambiguities can be attributed to BMI’s intrinsic inability to differentiate between fat and muscle mass and the inability to distinguish between generalized or centripetal obesity, leading to potential misclassifications and confounding results [23, 24].

Consequently, there is an urgent need for a more precise metric to capture the nuances of obesity and its potential cognitive impact. The Weight-adjusted-waist index (WWI), which combines waist circumference and weight, provides a refined measure of body fat distribution [24]. Considering abdominal obesity’s role in health conditions and BMI’s limitations in older adults, WWI may be critical for clarifying obesity-cognition links [24, 25].

Utilizing data from the National Health and Nutrition Examination Survey (NHANES) from 2011 to 2014, this study explores the association between WWI and cognitive function in adults aged 60 and above. By leveraging this expansive data repository, we aim to explore the association between WWI and cognitive function and further discern whether WWI can serve as a reliable predictor of cognitive decline.

## Methods

### Study population

This cross-sectional study aimed to explore the association between WWI and cognitive function in older adults using data from the NHANES database from 2011 to 2014. The NHANES comprehensive survey collects information through questionnaires and physical assessments, encompassing demographics, socioeconomic indicators, dietary patterns, and overall health status. The database also includes medical examinations featuring anthropometric measurements and laboratory evaluations. The NHANES survey protocol was approved by the National Center for Health Statistics (NCHS) Ethics Review Committee, with all participants providing written consent. Given the public nature of the NHANES database, our study did not require further ethical approval.

**Fig. 1:**
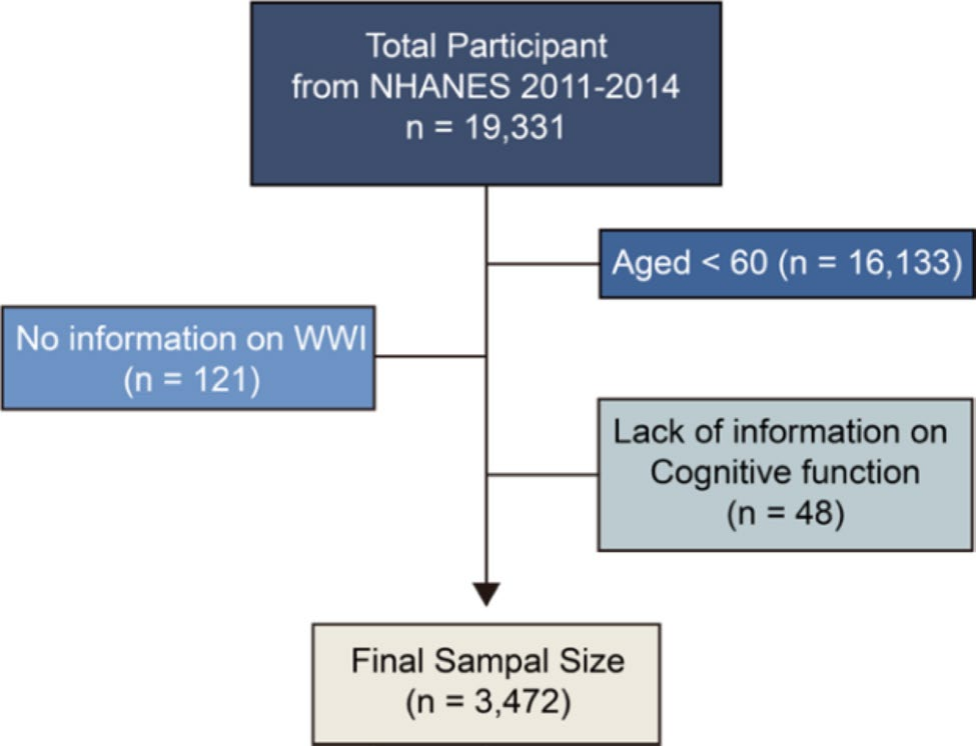
Flowchart of the sample selection process in this study

Our analysis incorporated 19,931 participants from the National Health and Nutrition Examination Survey (NHANES) cycles of 2011–2014. We excluded those below 60 years of age (*n* = 16,133). Additionally, we omitted individuals lacking essential data for computing the waist-to-weight ratio, specifically measurements of waist circumference and weight (*n* = 121) and lack of information on cognitive function (*n* = 48). Following these exclusions, the final cohort comprised 3,472 adults aged 60 and above, representing a significant nationally representative sample in the United States (Fig. 1).

### Explanatory variable

#### Weight-adjusted waist index

Weight-adjusted waist index (WWI) emerged as a novel and innovative marker of obesity that has responded to weight-independent abdominal obesity [23]. The weight-adjusted waist index (WWI), is derived from the waist circumference (WC in cm) divided by the square root of body weight (in kg). Anthropometric evaluations were conducted at the Mobile Examination Center (MEC) by trained technicians whose proficiency was periodically verified. Data on “Body Measures,” encompassing WC and weight, can be accessed on the NHANES website. Detailed methodologies are outlined in the NHANES manuals. The weight was measured using a Toledo digital scale, initially in pounds, and then converted to kilograms. For precision, participants were advised to wear minimal clothing during weighing. WC was ascertained using a measuring tape at designated anatomical sites. Elevated WWI values generally signify heightened obesity levels. For analytical purposes, participants were segmented into four quartiles based on WWI (Q1-Q4, Q1 ranged from 9.02 to 11.02, Q2 ranged from 11.02 to 11.51, Q3 ranged from 11.51 to 11.97 and Q4 ranged from 11.97 to 14.79), with WWI being central to our study.

### Outcome variables

#### Cognitive function

The National Health and Nutrition Examination Survey (NHANES) regularly assesses cognitive function in older adults aged 60 and above. During the household interview or the Mobile Examination Center evaluation, three tests determine cognitive capabilities: the Consortium to Establish a Registry for Alzheimer’s Disease Word Learning subtest (CERAD-WL) for assessing learning and memory; the Animal Fluency Test (AFT) for verbal fluency; and the Digit Symbol Substitution Test (DSST) for processing speed evaluation. Three principal parameters from CERAD-WL, AFT, and DSST delineate the cognitive performance of NHANES participants.

The CERAD-WL primarily assesses the immediate and delayed learning abilities for newly introduced verbal information, which falls under the memory sub-domain, a higher value in CERAD-WL indicates better memory performance [4, 26]. This test is organized into three consecutive learning trials followed by a delayed recall. Each trial presents 10 unrelated words, which participants either read aloud or repeat after the interviewer, depending on their literacy or visual capabilities. The words’ order is altered in each trial [27]. After the three learning trials and the other two tests (approximately 8–10 min later), participants were again asked to recall as many of the words as possible from the CERAD word list, and this score was used as a measure of delayed memory recall (CERAD-DR) resulting in an assessment of “Delayed Memory.” Final CERAD-WL scores encapsulate the outcomes from each learning trial, the delayed recall, and a count of intrusions, representing incorrect words not part of the original list. The CERAD is widely recognized in major epidemiological research, especially across racially and culturally diverse populations [26, 27].

An AFT is used to measure categorical verbal fluency, a subset of executive function [28]. Demonstrating its efficacy, the score from this test has been proficient at distinguishing individuals with normal cognitive function from those exhibiting mild or severe cognitive impairments or even disorders such as Alzheimer’s Disease. The test’s essence lies in the participant’s ability to enumerate as many animals as possible within a 60-second frame [29]. The AFT has been used in large-scale screenings and epidemiologic studies.

DSST, a Wechsler Adult Intelligence Scale (WAIS-III) segment, evaluates processing speed in conjunction with sustained attention and working memory [28]. Administered on a paper, participants are given a key pairing nine numbers with symbols, and they must correctly match symbols to numbers in adjacent boxes within 2 min. The total number of correct correspondences quantifies the performance. A preparatory trial precedes the main test to ensure participants’ understanding. Its significance is bolstered by its extensive application in broad screenings, epidemiological inquiries, and clinical research [30].

### Covariates

Based on our literature review, potential covariates that might confound the association between WWI and cognitive function were adjusted in our models [31–38]. Covariates adjusted in the current study mainly consisted of two categories: demographic status and medical conditions. Demographic covariates include age (years), gender (male/female), race (Mexican American / Other Hispanic / Non-Hispanic White /Non-Hispanic Black / Other), education status (Less than 9th grade / 9-11th grade / High school graduate / Some college /AA degree / College graduate / above / Other or Unknown) and smoking (yes/no). The medical condition includes diabetes (yes/no) and a history of stroke (yes/no), history of cardiovascular disease (yes/no), sleep disorder within a month (yes/no), history of depression (yes/no), currently on medication (yes/no).

### Statistical analysis

The mean of standard deviations and percentages or frequencies were used to represent continuous and categorical variables. Z-scores for CERAD-WL, CERAD-DR, AFT, and DSST were standardized using respective test means and standard deviations. Subsequently, global cognition z-scores were derived from these test-specific z-scores. The WWI score was transformed into a categorical variable (quartile) from a continuous variable. Weighted chi-squared tests and weighted t-tests were used to evaluate the differences among baseline variables. Weighted multiple regression analysis was used in three models to analyze the relationship between WWI and cognitive function, adjusting for pertinent covariates. The study used smooth curve fittings to explore the non-linear correlation. Subgroup analysis and interaction tests were used to investigate the relationship between WWI and cognitive function in different age groups of the elderly (including age, gender, stroke, and DM). All statistical analyses were performed on Python3, R version 4.3.2, and EmpowerStats (V5.0). A two-tailed p-value less than 0.05 was set as the standard of statistical significance.

### Machine learning pipeline

In this study, we conduct a machine learning analysis in our study to address a distinct scientific question related to the predictive power of the Waist-to-Weight Index (WWI) compared to traditional obesity indicators such as body mass index (BMI). The goal was not to develop a diagnostic model but rather to demonstrate that incorporating WWI as a variable can yield better predictive performance. This finding provides valuable insights for future research and suggests the potential inclusion of WWI as one of the indicators in training diagnostic models.

Machine learning (ML) models were trained based on the random forest classifier provided by the Caret package with features encompassing BMI or WWI and all covariables: age, gender, education level, race, smoking, diabetes, and stroke (i.e., two ML models were trained Model I incorporated all covariables with BMI, Model II comprised all covariables with WWI.). The models utilized a training set chosen at random, which accounted for 70% of the data and involved repeated sampling 10 times with 5-fold cross-validation, ntree was set to 1000. Their performance was evaluated on a separate validation set, making up the remaining 30%. Participants were categorized based on their cognitive functioning, as measured by their completion of the NHANES Cognitive Functioning Questionnaire (CFQ) (CFASTAT). CFQ is a series of assessments in NHANES (variable name prefix CFQ) that tests cognitive function, including: word learning and recall modules from the Consortium to Establish a Registry for Alzheimer’s Disease (CERAD), the Animal Fluency Test (AFT), and the Digit Symbol Substitution test (DSST). Exclusions from the Model included those who didn’t finish the test, often due to voluntary withdrawal. To ensure optimal AUROC scores on the validation set, the entire training and evaluation process was repeated 10 times with different random seeds.

### Code Availability

All code used in this study has been uploaded on GitHub and is publicly available [39].

## Results

### Baseline characteristics

In our investigation involving 3,472 respondents above 60, the mean age was 69.95 (6.94) years. Females constituted 51.14% of the sample. Among the participants, 306 (8.81%) had previously experienced a stroke, while 997 (28.7%) had been diagnosed with diabetes mellitus (DM). Notable disparities were observed across distinct WWI quartiles in gender, race/ethnicity, educational attainment, DM, stroke history, BMI, waist circumference, CFASTAT, CERAD-WL, AFT, and DSST. However, variations in CERAD-DR were not statistically significant (Table 1).

**Table 1:** Basic characteristics of participants by WWI quartile among US adults

Characteristics	WWI				*P*-value
	Q1 (*N* = 873) 9.02–11.02	Q2 (*N* = 872) 11.02–11.51	Q3 (*N* = 872) 11.51–11.97	Q4 (*N* = 873) 11.97–14.79	
**Age (years)**	67.50 ± 6.00	69.11 ± 6.60	69.87 ± 6.87	70.65 ± 7.05	< 0.01
60–70	520 (66.4%)	520 (66.4%)	385 (49.2%)	353 (45.1%)	
70–80	182 (23.2%)	228 (29.2%)	257 (32.9%)	254 (32.4%)	
Over 80	81 (10.3%)	119 (15.2%)	140 (17.9%)	176 (22.5%)	
**Gender**,** (%)**					< 0.01
Male	460 (58.7%)	434 (55.5%)	375 (48.0%)	266 (34.0%)	
Female	323 (41.3%)	348 (44.5%)	407 (52.0%)	517 (66.0%)	
**Race/ethnicity**,** (%)**					< 0.01
Mexican American	44 (5.6%)	63 (8.1%)	92 (11.8%)	95 (12.1%)	
Other Hispanic	323 (41.3%)	103 (13.2%)	76 (9.7%)	104 (13.3%)	
Non-Hispanic White	303 (38.7%)	335 (42.8%)	372 (47.6%)	392 (50.1%)	
Non-Hispanic Black	287 (36.7%)	197 (25.2%)	156 (19.9%)	113 (14.4%)	
Other	107 (13.7%)	84 (10.7%)	86 (11.0%)	79 (10.3%)	
**Education level**,** (%)**					< 0.01
Less than 9th grade	65 (8.3%)	92 (11.8%)	109 (13.9%)	175 (22.3%)	
9-11th grade	95 (12.1%)	129 (16.5%)	110 (14.1%)	118 (15.1%)	
High school graduate	175 (22.3%)	175 (22.4%)	178 (22.8%)	186 (23.8%)	
AA degree	213 (27.2%)	214 (27.4%)	227 (29.0%)	188 (24.0%)	
College graduate	235 (30.0%)	170 (21.7%)	158 (20.2%)	115 (14.7%)	
Other	0 (0.0%)	2 (0.3%)	0 (0.0%)	1 (0.1%)	
**Diabetes**,** (%)**					< 0.01
Yes	128 (16.3%)	201 (25.7%)	245 (31.3%)	311 (39.7%)	
No	655 (83.7%)	581 (74.3%)	537 (68.7%)	472 (60.3%)	
**Stroke**,** (%)**					< 0.01
Yes	48 (6.1%)	56 (7.2%)	51 (6.5%)	87 (11.1%)	
No	735 (93.9%)	726 (92.8%)	731 (93.5%)	696 (88.9%)	
CVD					
Yes	50(6.42%)	67(8.63%)	68(8.73%)	100(12.89%)	< 0.01
No	729(93.58%)	709(91.37%)	711(91.27%)	676(87.11%)	
**Sleep disorder**,** (%)**					
Yes	52(6.65%)	94(12.04%)	684(87.47%)	119(15.24%)	< 0.01
No	730(93.35%)	687(87.96%)	98(12.53%)	662(84.76%)	
**On medication**,** (%)**					
Yes	607(77.52%)	663(84.78%)	690(88.46%)	713(91.18%)	< 0.01
No	176(22.48%)	119(15.22%)	90(11.45%)	69(8.82%)	
**Depression**					
Yes	41(5.44%)	59(7.77%)	60(8.04%)	78(10.47%)	0.06
No	713(94.56%)	700(92.23%)	686(91.96%)	667(89.53%)	
**Waist Circumference**,** (cm)**	117.72 ± 12.51	104.59 ± 8.56	97.38 ± 8.51	87.82 ± 9.13	< 0.01
**BMI**	35.53 ± 6.37	29.91 ± 3.83	27.00 ± 3.34	23.48 ± 3.20	< 0.01
**CFASTAT**					0.05
Yes	62 (7.9%)	57 (7.3%)	68 (8.7%)	86 (11.0%)	
No	721 (92.1%)	725 (92.7%)	714 (91.3%)	697 (89.0%)	
**CERAD - WL**	25.41 ± 6.62	24.71 ± 6.82	24.52 ± 6.64	23.92 ± 6.94	< 0.01
**CERAD - DR**	6.14 ± 2.26	5.94 ± 2.25	5.93 ± 2.25	5.93 ± 2.39	0.232
**AFT**	17.04 ± 5.70	16.71 ± 5.53	16.60 ± 5.40	15.72 ± 5.43	< 0.01
**DSST**	47.36 ± 15.97	47.17 ± 17.24	45.56 ± 16.82	45.03 ± 18.21	< 0.01

Mean ± SD for continuous variables: the P value was calculated by the weighted linear regression model; (%) for categorical variables: the P value was calculated by the weighted chi-square test. Abbreviation: WWI, Weight-Adjusted-Waist Index, BMI, body mass index; CERAD-WL/DR, the Consortium to Establish a Registry for Alzheimer’s Disease Word Learning subtest for assessing learning and memory; AFT, the Animal Fluency Test for verbal fluency; and DSST, the Digit Symbol Substitution Test; Q, quartile

### Association between WWI and cognitive function

Table 2 shows the results of multivariate regression analyses for three models. A significant negative linear relationship between WWI and cognitive function measured by CERAD-WL [-0.96 (-1.30, -0.62)], CERAD-DR [-0.28 (-0.40, -0.16)], AFT [-0.77 (-1.05, -0.49)], and DSST [-3.67 (-4.55, -2.79) in the unadjusted model. The negative correlation between WWI and cognitive function measured by CERAD-WL [-0.40 (-0.73, -0.06)], AFT [-0.28 (-0.55, -0.01) ], and DSST [-1.66 (-2.37, -0.96) remains significant even after adjusting for all covariates. However, after adjusting for all covariates, this significant negative correlation between WWI and CERAD-DR became insignificant in model 2 [-0.09 (-0.21, 0.03)].

We further investigated the association between WWI and cognitive functions after transforming WWI into categorical variables. In the fully adjusted model, the significant negative association between WWI and cognitive function measured by CERAD-WL, AFT, and DSST persisted (all P for trend < 0.01). Using participants in the lowest quartile of the WWI as the reference group, participants in the highest quartile of the WWI (11.97–14.79) had a significant decrease in CERAD-WL scores of 1.35 [-1.35 (-2.58, -0.12)], AFT scores of 0.69 [-0.69 (-1.64, 0.27) ], and DSST scores of 2.15 [-2.15 (-4.79, 0.50)].

**Table 2:** Associations between WWI with CERAD – WL, CERAD -DR, AFT, and DSST

WWI	CERAD - WL	CERAD -DR	AFT	DSST
	β (95% CI) pvalue	β (95% CI) pvalue	β (95% CI) pvalue	β (95% CI) pvalue
**Crude Model (Model 1)**				
**Continuous**	**-0.96 (-1.30**,** -0.62) < 0.01**	**-0.28 (-0.40**,** -0.16) < 0.01**	**-0.77 (-1.05**,** -0.49) < 0.01**	**-3.67 (-4.55**,** -2.79) < 0.01**
Categories				
Quartile 1	Reference	Reference	Reference	Reference
Quartile 2	-0.72 (-1.41, -0.04) 0.03	-0.18 (-0.42, 0.06) 0.15	-0.26 (-0.83, 0.30) 0.36	-2.31 (-4.06, -0.56) 0.01
Quartile 3	-0.91 (-1.60, -0.22) 0.01	-0.26 (-0.50, -0.02) 0.04	-0.44 (-1.00, 0.13) 0.13	-1.76 (-3.52, 0.00) 0.05
Quartile 4	-1.51 (-2.20, -0.82) < 0.01	-0.35 (-0.59, -0.12) < 0.01	-1.27 (-1.84, -0.70) < 0.01	-7.33 (-9.11, -5.54) < 0.01
*P for tend*	0.02	0.83	<0.01	< 0.01
**Partly adjusted Model (Model 2)**				
**Continuous**	**-0.46 (-0.78**,** -0.13) < 0.01**	**-0.11 (-0.23**,** 0.00) 0.05**	**-0.34 (-0.61**,** -0.08) 0.01**	**-1.95 (-2.64**,** -1.26) < 0.01**
Categories				
Quartile 1	Reference	Reference	Reference	Reference
Quartile 2	-0.42 (-1.08, 0.23) 0.20	-0.06 (-0.29, 0.17) 0.60	0.00 (-0.55, 0.56) 0.99	-1.52 (-3.20, 0.16) 0.07
Quartile 3	-0.52 (-1.18, 0.15) 0.12	-0.10 (-0.33, 0.13) 0.40	0.03 (-0.54, 0.59) 0.93	-0.80 (-2.50, 0.90) 0.35
Quartile 4	-1.32 (-2.00, -0.64) 0.01	-0.35 (-0.59, -0.12) < 0.01	-0.66 (-1.24, -0.09) 0.02	-6.73 (-8.48, -4.98) < 0.01
*P for tend*	0.02	0.65	<0.01	< 0.01
**Fully adjusted Model (Model 3)**				
**Continuous**	**-1.77 (-3.03**,** -0.50) < 0.01**	**-0.55 (-1.00**,** -0.10) 0.01**	**-0.78 (-1.44**,** -0.13) < 0.01**	**-1.87 (-2.76**,** -0.98) < 0.01**
Categories				
Quartile 1	Reference	Reference	Reference	Reference
Quartile 2	-0.03 (-3.27, 3.32) 0.98	0.49 (-0.66, 1.64) 0.40	0.44 (-2.18, 3.06) 0.74	6.62 (0.34, 12.89) 0.03
Quartile 3	0.10 (-3.21, 3.40) 0.95	0.09 (-1.10, 1.28) 0.88	-1.48 (-4.22, 1.26) 0.29	-1.54 (-9.01, 5.92) 0.68
Quartile 4	-1.35 (-2.58, -0.12) 0.03	-0.29 (-0.74, 0.16) 0.21	-0.69 (-1.64, 0.27) 0.16	-2.15 (-4.79, 0.50) 0.11
*P for tend*	< 0.01	0.21	0.01	0.05

Model 1: No covariates were adjusted. Model 2: age, gender, and race were adjusted. Model 3: age, gender, race, education level, drug use, sleep disorder, CVD, depress were adjusted. WWI, Weight-adjusted Waist Index, CERAD-WL, Consortium to Establish a Registry for Alzheimer’s disease Word Learning subtest, CERAD-DR, Consortium to Establish a Registry for Alzheimer’s Disease Delayed Recall, AFT, Animal Fluency Test, DSST, Digit Symbol Substitution test

### Subgroup analysis

To examine the association between WWI and cognitive function in different populations, we conducted subgroup analyses examining variables including gender, age, smoking status, diabetes mellitus (DM), and stroke history. Focusing first on gender, we observed a consistent negative association between WWI and CERAD-WL, AFT, and DSST across all genders. However, this correlation was notably stronger in male participants than in females. When segregating participants by age, the findings suggested that the negative association between WWI and CERAD-WL/DR, AFT, and DSST was consistent across all age groups. Nevertheless, this negative correlation diminished with increasing age. Finally, a more pronounced negative correlation between WWI and CERAD-WL/DR, AFT, and DSST was evident among participants without a history of smoking, DM, or stroke (Table 3).

**Table 3:** Subgroup analysis of the association between dietary WWI and CERAD-WL, CERAD-DR, AFT, and DSST

Subgroup	CERAD-WL	CERAD-DR	AFT	DSST
	β/OR (95%CI) pvalue	β/OR (95%CI) pvalue	β/OR (95%CI) pvalue	β/OR (95%CI) pvalue
**Smoke**				
No	-1.23 (-1.71, -0.75) < 0.01	-0.33 (-0.50, -0.16) 0.01	-0.70 (-1.09, -0.30) < 0.01	-3.74 (-4.97, -2.51) < 0.01
Yes	-0.46 (-0.93, 0.00) 0.05	-0.11 (-0.28, 0.05) 0.17	-0.19 (-0.60, 0.22) 0.36	-2.89 (-4.12, -1.66) < 0.01
*P for interaction*	*< 0.01*	*0.02*	*< 0.01*	*< 0.01*
**Diabetes**				
No	-0.40 (-0.79, -0.01) 0.04	-0.09 (-0.23, 0.05) 0.19	-0.24 (-0.57, 0.08) 0.14	-1.71 (-2.55, -0.88) < 0.01
Yes	-0.33 (-0.98, 0.32) 0.33	-0.05 (-0.28, 0.18) 0.69	-0.39 (-0.88, 0.10) 0.11	-1.33 (-2.64, -0.02) 0.05
*P for interaction*	*0.02*	*0.14*	*0.04*	*< 0.01*
**Stroke**				
No	-0.31 (-0.65, 0.04) 0.08	-0.08 (-0.20, 0.05) 0.23	-0.26 (-0.55, 0.02) 0.06	-1.49 (-2.21, -0.76) < 0.01
Yes	-1.55 (-2.84, -0.27) 0.01	-0.29 (-0.72, 0.13) 0.18	-0.64 (-1.65, 0.37) 0.21	-3.86 (-6.62, -1.10) < 0.01
*P for interaction*	*0.02*	*0.13*	*0.04*	*< 0.01*
**Gender**				
Male	-1.18 (-1.68, -0.69) < 0.01	-0.12 (-0.30, 0.06) 0.19	-0.42 (-0.84, 0.01) 0.06	-2.30 (-3.32, -1.28) < 0.01
Female	-5.05 (-6.28, -3.82) < 0.01	-0.06 (-0.22, 0.10) 0.48	-0.15 (-0.50, 0.20) 0.41	-1.09 (-2.07, -0.10) 0.03
*P for interaction*	*< 0.01*	*0.13*	*0.04*	*< 0.01*
**Age**				
60 ~ 70	-0.43 (-0.87, 0.01) 0.03	-0.05 (-0.21, 0.10) 0.49	-0.35 (-0.73, 0.03) 0.07	-1.80 (-2.79, -0.82) < 0.01
70 ~ 80	-1.00 (-1.64, -0.36) < 0.01	-0.32 (-0.55, -0.08) < 0.01	-0.21 (-0.73, 0.31) 0.43	-2.69 (-4.01, -1.37) < 0.01
80~	0.20 (-0.67, 1.08) 0.65	-0.01 (-0.31, 0.30) 0.97	-0.45 (-1.01, 0.11) 0.11	-1.27 (-2.89, 0.34) 0.12
*P for interaction*	*< 0.01*	*0.05*	*0.01*	*< 0.01*

### Non-linear correlation between WWI and cognitive function

**Fig. 2:**
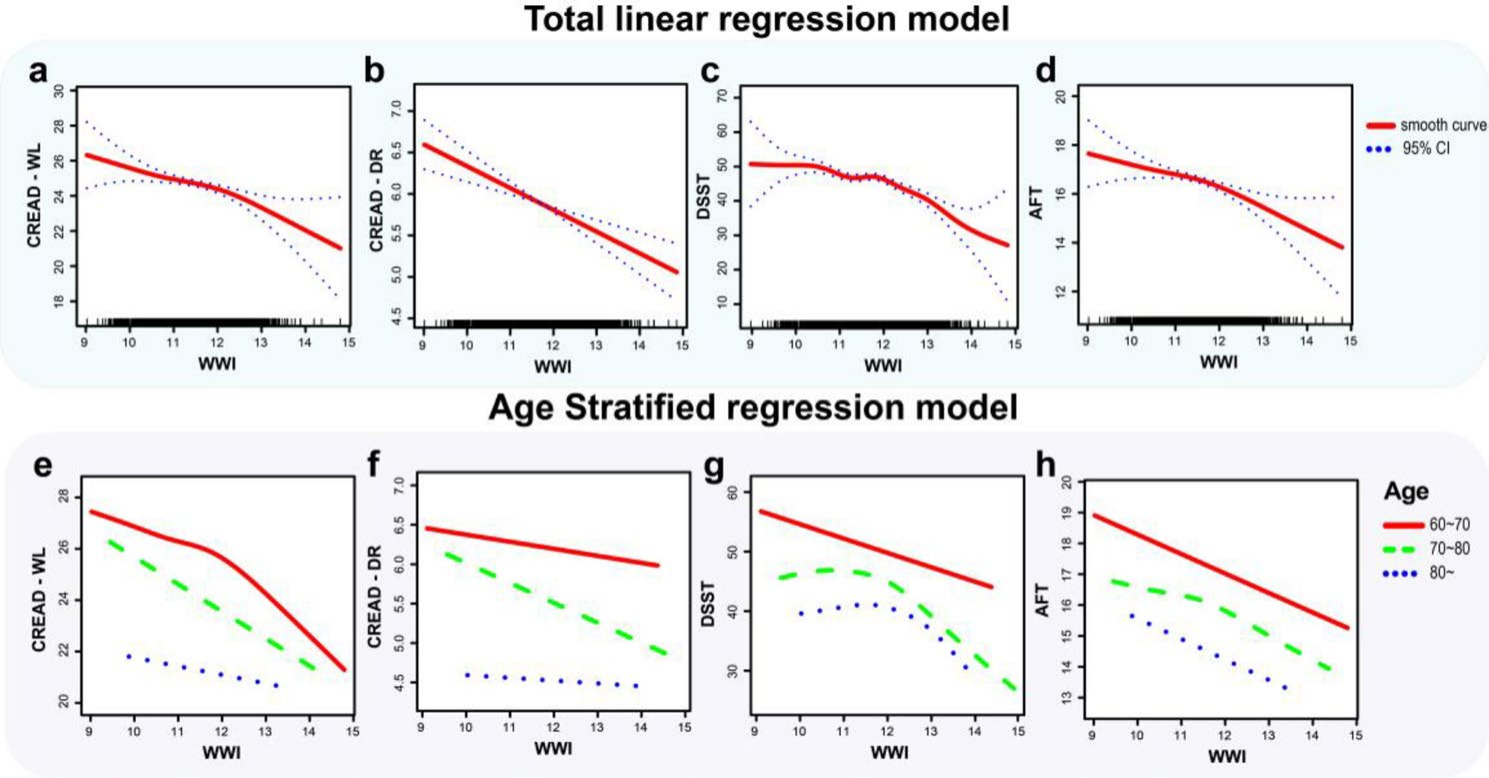
**(a-d)** Total linear regression model of WWI versus CERAD-WL, CERAD-DR, DSST, AFT, the Model was adjusted by age, gender, race, smoke, diabetes, and education level. **(e-h)** The age-stratified linear regression model of WWI versus CERAD-WL, CERAD-DR, DSST, and AFT was adjusted by age, gender, race, smoking, diabetes, and education level

To understand the correlation between WWI and cognitive function, we performed a smoothed curve-fitting analysis to explore the potential non-linear association between WWI and CERAD-WL/DR, DSST, and AFT and determine if a threshold effect exists. The threshold effect model demonstrated a non-linear correlation between WWI and CERAD-WL (LLR < 0.05). The inverse association between WWI and CERAD-WL became more pronounced when WWI surpassed 12.25, resulting in an effect size of (OR = -2.57, 95% CI: -4.32 to -0.82). Likewise, the negative correlations between WWI and both AFT and DSST intensified at WWI values exceeding 11.54 and 11.66, respectively. The effect sizes were (OR = -0.98, 95% CI: -1.49 to -0.47, LLR < 0.01) and (OR = -5.82, 95% CI: -7.46 to -4.19). Conversely, a linear relationship was observed between WWI and CERAD-DR (LLR = 0.14). Further details can be found in Table 4; Fig. 2a-d.

To further assess the influence of WWI on cognitive function, we stratified the Model by age (refer to Table 4; Fig. 2e-g). We discovered that for CERAD-WL, its non-linear characteristic was primarily contributed by individuals aged 60–70 years (Inflection point = 12.19, LLR < 0.01). CERAD-WL decreased monotonically with WWI in other age groups, suggesting a linear trend. For DSST, the non-linear feature was significant across all age groups, especially those aged 70–80 and above 80. The inflection points for these age groups were 11.84 (LLR = 0.03), 11.65 (LLR < 0.01), and 12.32 (LLR < 0.01), respectively.

**Table 4:** Non-linear correlation between WWI and CERAD-WL, CERAD-DR, AFT, and DSST

WWI	CERAD-WL	CERAD-DR	AFT	DSST
	β (95% CI)	β (95% CI)	β (95% CI)	β (95% CI)
**Total**				
Fitting by the standard linear Model	-0.69 (-1.03, -0.34)	-0.17 (-0.29, -0.05)	-0.56 (-0.84, -0.28)	**-2.88 (-3.68**,** -2.07)**
Fitting by the two-piecewise linear Model				
Inflection point (K)	12.25	12.1	11.54	11.66
< K-segment effect	-0.45 (-0.87, -0.03)	-0.11 (-0.26, 0.05)	-0.16 (-0.66, 0.34)	-0.75 (-2.05, 0.55)
> K-segment effect	-1.89 (-3.16, -0.62)	-0.41 (-0.79, -0.04)	-0.98 (-1.49, -0.47)	-5.82 (-7.46, -4.19)
*Log likelihood ratio*	0.05	0.19	0.05	< 0.01
**Age Stratification**				
**60 ~ 70**				
Fitting by the standard linear Model	-0.49 (-0.92, -0.06)	-0.09 (-0.24, 0.07)	-0.41 (-0.78, -0.04)	-1.97 (-2.94, -1.01)
Fitting by the two-piecewise linear Model				
Inflection point (K)	12.19	11.75	12.59	11.84
< K-segment effect	-0.14 (-0.66, 0.38)	0.04 (-0.19, 0.26)	-0.31 (-0.71, 0.08)	-0.97 (-2.31, 0.36)
> K-segment effect	-2.57 (-4.32, -0.82)	-0.35 (-0.73, 0.04)	-1.97 (-4.47, 0.53)	-4.70 (-7.41, -1.99)
*log likelihood ratio*	< 0.01	0.14	0.22	0.03
**70 ~ 80**				
Fitting by the standard linear Model	-0.96 (-1.57, -0.34)	-0.31 (-0.54, -0.09)	-0.33 (-0.83, 0.18)	-3.11 (-4.40, -1.83)
Fitting by the two-piecewise linear Model				
Inflection point (K)	11.67	11.67	11.47	11.65
< K-segment effect	-1.73 (-2.78, -0.67)	-0.61 (-0.99, -0.22)	0.21 (-0.81, 1.23)	-5.64 (-8.10, -3.17)
> K-segment effect	-0.02 (-1.23, 1.19)	0.05 (-0.39, 0.48)	-0.74 (-1.59, 0.10)	-4.72 (-8.65, -0.79)
*Log likelihood ratio*	0.08	0.06	0.22	< 0.01
**80~**				
Fitting by the standard linear Model	0.11 (-0.75, 0.98)	-0.03 (-0.34, 0.27)	-0.44 (-1.00, 0.11)	-1.44 (-3.04, 0.15)
Fitting by the two-piecewise linear Model				
Inflection point (K)	13.03	13.03	12.27	12.32
< K-segment effect	0.34 (-0.61, 1.29)	0.07 (-0.26, 0.40)	-0.04 (-0.80, 0.73)	0.44 (-1.72, 2.60)
> K-segment effect	-5.96 (-16.29, 4.37)	-2.75 (-6.38, 0.88)	-1.74 (-3.48, 0.01)	-8.55 (-15.19, -1.91)
*Log likelihood ratio*	0.24	0.13	0.12	< 0.01

Age, gender, race, education level, and BMI were adjusted. Abbreviation: WWI, Weight-adjusted Waist Index, CERAD-WL, Consortium to Establish a Registry for Alzheimer’s disease Word Learning subtest, CERAD-DR, Consortium to Establish a Registry for Alzheimer’s Disease Delayed Recall, AFT, Animal Fluency Test, DSST, Digit Symbol Substitution test

### WWI as a robust indicator for binary machine learning models

Two machine-learning models were developed using the data we amassed based on the random forest classifier. The features integrated for training encompassed all covariables along with either WWI or BMI. Specifically, Model I incorporated all covariables with BMI, and Model II comprised all covariables with WWI. Comprehensive training methodologies are elaborated in the “Materials and Methods” section. Participant categorization relied on their cognitive function as assessed by completing the NHANES CFQ questionnaire. Those not finalizing the test, often due to reasons like voluntary withdrawal, were excluded.

**Fig. 3:**
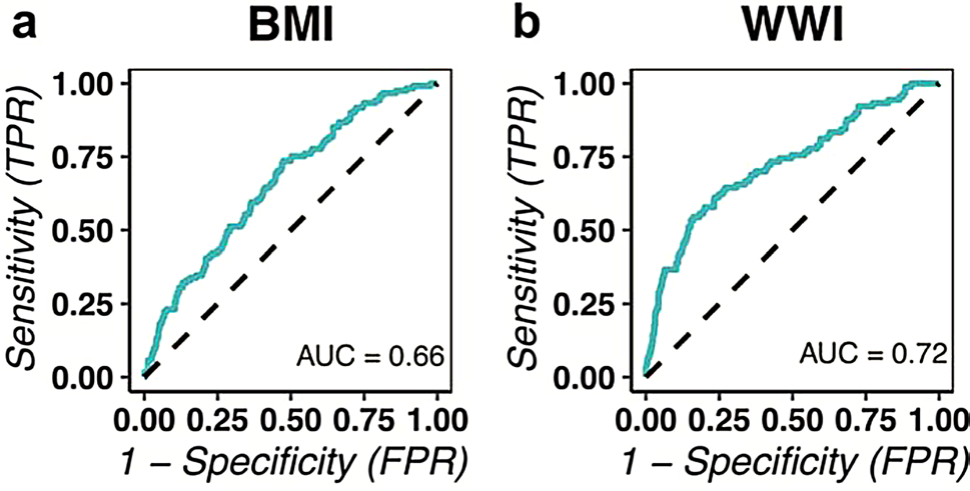
ROC of Binary ML models trained based on random forest. **(a)** ROC of Model I, feature included all covariables and BMI. **(b)** ROC of Model II, the feature consists of all covariables and WWI

The results highlighted an AUROC of 0.66, and for the BMI-inclusive Model, 0.72 for the WWI-inclusive Model. This implies a potentially enhanced predictive capacity of WWI for cognitive function over BMI (Fig. 3).

## Discussion

In this cross-sectional analysis of NHANES data, we discovered a consistent negative association between WWI and multiple measures of cognitive function, including learning, memory, verbal fluency, and processing speed, in adults aged 60 and above. Our findings further revealed non-linear relationships characterized by threshold effects between WWI and certain cognitive tests.

Due to the prevalence and significant health risks of obesity, an increasing number of indicators are being used to assess obesity, particularly focusing on the recognized harmfulness of visceral fat. While body mass index (BMI) is commonly used as an obesity index, numerous studies have investigated the relationship between BMI and cognitive function, yet their findings are inconsistent [40–42]. While some studies suggest a negative correlation between obesity and cognitive abilities, others reveal no significant association, and a few even indicate a positive relationship [43]. For example, a substantial cohort study by Kim et al. in Korea observed that a higher BMI often correlated with a decreased risk of cognitive decline in the middle-aged and elderly population [44]. Conversely, Lackner et al. identified BMI as a potential risk factor for cognitive deficits [45]. This is why many studies have proposed the “obesity paradox” phenomenon regarding body mass index (BMI) [23, 46, 47], which suggests that individuals who are relatively overweight have better cognitive function compared to those within the normal range of BMI.

The divergence in these outcomes may arise from the inherent shortcomings of the BMI measure [13]. While BMI, determined by weight relative to height, offers a broad weight gauge, it doesn’t distinguish between fat and muscle mass [48]. Consequently, two individuals with the same BMI can exhibit vast differences in their body compositions, thus presenting varied health implications [49]. Notably, as individuals age, the body fat-to-muscle ratio shifts, a nuance not captured by BMI [50].

To delve deeper into the association between obesity and cognitive function with greater precision and circumvent the ambiguities associated with BMI, we opted to utilize WWI as an innovative obesity index [51]. Waist-to-weight index (WWI) is a body measurement index used to assess central obesity, and it is defined as waist circumference divided by the square of body weight [23]. It reflects both fat content and muscle mass, even across different body mass index categories [50, 52]. Weight-adjusted waist index (WWI) is a body measurement index for central obesity, defined as waist circumference divided by the square of body weight [23]. It reflects both fat content and muscle mass, even across different body mass index categories [50, 52]. WWI takes into account waist circumference and adjusts it relative to weight, thereby presenting a more holistic view of body fat distribution [52]. We aim to provide more lucid and exact interpretations in this complex area by employing WWI. Based on the machine learning models trained separately with BMI and WWI, we found that WWI might be better than BMI in predicting cognitive functions (Fig. 3).

As a distinctive indicator of abdominal obesity, WWI accounts for weight status, conferring greater precision than waist circumference alone [23, 52] The value of the Waist-to-Weight Index (WWI) in predicting other well-established obesity-related outcomes has been increasingly supported in recent years. The waist-to-weight index (WWI) adjusted for body weight has been shown to reflect centrally distributed obesity independent of overall body weight. Previous research has indicated that WWI is an important predictor of cardiovascular disease incidence and mortality, surpassing other indices such as body mass index (BMI), a body shape index (ABSI), and waist-to-height ratio (WHtR) [23, 46]. Additionally, WWI has been found to be a better predictor of hypertension incidence compared to BMI and waist circumference (WC) [51]. Weight-adjusted waist index (WWI) has been confirmed to reflect weight-independent central obesity. Previous studies have shown that WWI is an important indicator for predicting the incidence and mortality of cardiovascular diseases, surpassing body mass index (BMI), adiposity-based index (ABSI), and waist-to-height ratio (WHtR) [23, 46]. Additionally, WWI has been found to be a better predictor of hypertension incidence compared to BMI and waist circumference (WC) [46]. The accumulating body of research supports the potential of WWI as a valuable tool for predicting various obesity-related outcomes. By incorporating waist circumference and weight into a single index, WWI provides a comprehensive and practical measure of obesity that can aid in assessing an individual’s risk for a range of health conditions. However, research focused on WWI and cognitive function remains limited. Our results align with and extend existing evidence by demonstrating the utility of WWI as a predictor of cognitive decline. To our knowledge, we are the first to investigate the association between WWI and cognitive function.

We found the strongest negative correlations occurred between WWI and CERAD-WL and DSST, both of which assess learning/memory and processing speed. This coincides with research pinpointing memory and processing speed as cognitive domains especially vulnerable to the effects of adiposity [53–55]. The mechanisms underlying the link between obesity and cognition are complex and multifaceted. While the exact processes are still being explored, several mechanisms have been proposed to explain this relationship [56]. One key mechanism is inflammation. Obesity is associated with chronic low-grade inflammation, as increased adipose tissue produces pro-inflammatory cytokines and other molecules. This inflammatory state can lead to oxidative stress and damage to brain cells, ultimately affecting cognitive function [57]. Another mechanism is insulin resistance. Obesity often coincides with insulin resistance, where cells become less responsive to the effects of insulin. Since insulin plays a crucial role in brain function and glucose metabolism, impaired insulin signaling in the brain can result in reduced neuronal function and cognitive deficits(57). Vascular factors also play a role. Obesity is linked to various cardiovascular risk factors, including hypertension, dyslipidemia, and atherosclerosis [58]. These factors can contribute to reduced blood flow and compromised vascular health in the brain [58]. Impaired cerebral blood flow and vascular dysfunction have been associated with cognitive decline [59]. Hormonal dysregulation is another contributing factor. Obesity is characterized by alterations in hormone levels, such as leptin and adiponectin. Dysregulation of these hormones can impact brain function and synaptic plasticity, which are essential for learning and memory processes [60]. Additionally, the central nervous system effects of obesity should be considered. Adipose tissue produces hormones and molecules called adipokines, some of which, like leptin and adiponectin, can cross the blood-brain barrier and influence neuronal function [61]. Disruptions in the signaling of these adipokines may affect cognitive processes [61]. It is important to note that these mechanisms are interconnected, and the relationship between obesity and cognitive functioning is likely influenced by a combination of these factors. However, the specific mechanisms and their relative contributions are still areas of active research.

Interestingly, our curve-fitting analyses revealed non-linear threshold associations for CERAD-WL, AFT, and DSST but not CERAD-DR. A possible explanation is that CERAD-DR specifically measures long-term retention, which may be less impacted by adiposity until more severe impairment occurs. The inflection points for CERAD-WL (12.25) and DSST (11.66) aligned closely with the cut-offs proposed for defining central obesity using WWI [33]. This lends further support to the utility of these WWI thresholds.

We also found that the relationship between WWI and cognition was more robust in men and weakened with older age. These trends have been noted in prior obesity-cognition research [28, 61–63] and may reflect the disproportionate contribution of cardiovascular risks versus neurodegeneration in younger versus older generations. Additionally, lower cognitive reserve among women and differential sex hormone profiles may confer relative protection in women [64–66].

However, several limitations of this study warrant attention. The cross-sectional nature of our design inhibits the determination of causal relationships, necessitating longitudinal assessments to ascertain temporal patterns [67]. The possibility of residual confounding remains, especially considering unaccounted variables like physical activity. Moreover, the absence of brain imaging data restricts our ability to correlate WWI with structural degeneration.

Nevertheless, this investigation notably enriches existing literature on BMI and waist circumference metrics. The potential of WWI thresholds in identifying cognitive vulnerabilities became evident. These nationally representative findings suggest that an elevated WWI correlates with diminished cognitive health in older individuals. Addressing abdominal obesity might yield cognitive advantages.

In conclusion, our study found higher WWI, an indicator of abdominal obesity, is associated with poorer learning, memory, verbal fluency, and processing speed in a national sample of older Americans. These findings highlight the potential utility of WWI as a modifiable risk factor for cognitive aging.

### Electronic supplementary material

Below is the link to the electronic supplementary material.

Supplementary Material 1

## Data Availability

All data analyzed in the current study are freely accessible on the NHANES website [68].

## Abbreviations

WWI: Weight-Adjusted-Waist Index
BMI: Body Mass Index
NHANES: National Health and Nutrition Examination Survey
NCHS: National Center for Health Statistics
WC: Waist Circumference
MEC: Mobile Examination Center
CERAD: Consortium to Establish a Registry for Alzheimer’s Disease
CERAD-WL: Consortium to Establish a Registry for Alzheimer’s Disease Word Learning
AFT: Animal Fluency Test
DSST: Digit Symbol Substitution Test
CERAD-DR: Consortium to Establish a Registry for Alzheimer’s Diseases Delayed Memory Recall
WAIS-III: Wechsler Adult Intelligence Scale; ML: Machine learning
