# Supplementary material for: The association between Weight-adjusted-Waist Index (WWI) and cognitive function in older adults: a cross-sectional NHANES 2011–2014 study
Source: BMC Public Health. 2024 Aug 8;24:2152. doi: 10.1186/s12889-024-19332-w (PMC11308487; doi:10.1186/s12889-024-19332-w)
Supplement: Supplementary file 1 — Supplementary Material 1 [file 12889_2024_19332_MOESM1_ESM.docx]

**Table. s1 :** Associations between BMI with CERAD – WL, CERAD -DR, AFT, and DSST.

| **WWI** | **CERAD - WL** | **CERAD -DR** | **AFT** | **DSST** |
| --- | --- | --- | --- | --- |
|  | β (95% CI) pvalue | β (95% CI) pvalue | β (95% CI) pvalue | β (95% CI) pvalue |
| **Crude Model (Model 1)** |  |  |  |  |
| **Continuous** | **0.06 (0.03, 0.10) <0.01** | **0.02 (0.01, 0.04) <0.01** | **0.04 (0.01, 0.07) 0.02** | **0.01 (-0.09, 0.11) 0.85** |
| Categories |  |  |  |  |
| Quartile 1 | Reference | Reference | Reference | Reference |
| Quartile 2 | 0.06 (-0.62, 0.74) 0.86 | -0.07 (-0.31, 0.17) 0.60 | 0.34 (-0.23, 0.91) 0.24 | 1.00 (-0.79, 2.79) 0.27 |
| Quartile 3 | 0.29 (-0.38, 0.96) 0.40 | 0.03 (-0.21, 0.26) 0.83 | 0.43 (-0.13, 0.99) 0.13 | 0.43 (-1.34, 2.20) 0.63 |
| Quartile 4 | 1.07 (0.40, 1.75) <0.01 | 0.36 (0.12, 0.60) <0.01 | 0.59 (0.02, 1.15) 0.04 | 0.28 (-1.50, 2.06) 0.76 |
| *P for tend* | <0.01 | <0.01 | 0.046 | 0.97 |
| **Partly adjusted Model (Model 2)** |  |  |  |  |
| **Continuous** | **0.02 (-0.02, 0.06) 0.26** | **0.01 (0.00, 0.03) 0.04** | **0.01 (-0.02, 0.04) 0.66** | **-0.04 (-0.13, 0.05) 0.39** |
| Categories |  |  |  |  |
| Quartile 1 | Reference | Reference | Reference | Reference |
| Quartile 2 | 0.47 (-0.16, 1.11) 0.1436 | 0.10 (-0.13, 0.32) 0.41 | 0.13 (-0.40, 0.66) 0.63 | 2.18 (0.65, 3.71) <0.01 |
| Quartile 3 | 0.47 (-0.16, 1.10) 0.1461 | 0.15 (-0.08, 0.37) 0.21 | 0.07 (-0.47, 0.60) 0.81 | 1.43 (-0.10, 2.96) 0.07 |
| Quartile 4 | 0.60 (-0.05, 1.25) 0.0694 | 0.29 (0.06, 0.52) 0.01 | 0.09 (-0.45, 0.63) 0.75 | 0.07 (-1.49, 1.63) 0.93 |
| *P for tend* | 0.10 | 0.01 | 0.83 | 0.61 |
| **Fully adjusted Model (Model 3)** |  |  |  |  |
| **Continuous** | **0.03 (-0.01, 0.07) 0.10** | **0.02 (0.00, 0.03) 0.01** | **0.02 (-0.01, 0.05) 0.24** | **0.02 (-0.06, 0.10) 0.60** |
| Categories |  |  |  |  |
| Quartile 1 | Reference | Reference | Reference | Reference |
| Quartile 2 | 0.34 (-0.28, 0.96) 0.2789 | 0.07 (-0.15, 0.29) 0.55 | 0.06 (-0.45, 0.57) 0.82 | 1.84 (0.51, 3.17) <0.01 |
| Quartile 3 | 0.61 (-0.01, 1.23) 0.0551 | 0.20 (-0.03, 0.42) 0.09 | 0.24 (-0.27, 0.76) 0.35 | 2.06 (0.73, 3.40) <0.01 |
| Quartile 4 | 0.71 (0.06, 1.35) 0.0318 | 0.33 (0.10, 0.56) <0.01 | 0.21 (-0.33, 0.74) 0.44 | 0.73 (-0.66, 2.11) 0.30 |
| *P for tend* | 0.03 | <0.01 | 0.38 | 0.48 |

Model 1: No covariates were adjusted. Model 2: age, gender, and race were adjusted. Model 3: age, gender, race, and education level, drug use, sleep disorder, CVD, depress were adjusted. WWI, Weight-adjusted Waist Index, CERAD-WL, Consortium to Establish a Registry for Alzheimer’s disease Word Learning subtest, CERAD-DR, Consortium to Establish a Registry for Alzheimer’s Disease Delayed Recall, AFT, Animal Fluency Test, DSST, Digit Symbol Substitution test.
